# Acute kidney injury and outcomes in hospitalized children with autoimmune rheumatic disease

**DOI:** 10.1186/s13052-025-01862-7

**Published:** 2025-02-07

**Authors:** Chien-Hung Lin, Wen-Sheng Liu, Chuan Wan, Hsin-Hui Wang

**Affiliations:** 1 Division of Pediatric Immunology and Nephrology, Department of Pediatrics, Taipei Veterans General Hospital, No.201, Sec. 2, Shipai Rd., Beitou District, 11201 Taipei, Taiwan; 2 School of Medicine, National Yang Ming Chiao Tung University, Taipei, Taiwan; 3 College of Science and Engineering, Fu Jen Catholic University, New Taipei, Taiwan; 4 Division of Nephrology, Department of Medicine, Taipei City Hospital Zhongxing Branch, Taipei, Taiwan; 5 Institute of Food Safety and Health Risk Assessment, National Yang Ming Chiao Tung University, Hsinchu, Taiwan; 6 Department of Special Education, University of Taipei, Taipei, Taiwan; 7 Department of Pediatrics, Taipei City Hospital, Zhongxing Branch, Taipei, Taiwan; 8 Institute of Clinical Medicine, National Yang Ming Chiao Tung University, Taipei, Taiwan; 9 Institute of Emergency and Critical Care Medicine, National Yang Ming Chiao Tung University, Taipei, Taiwan

**Keywords:** Acute kidney injury (AKI), Autoimmune rheumatic diseases (ARDs), Dialysis, Children, Nationwide Inpatient Sample (NIS)

## Abstract

**Background:**

Autoimmune rheumatic diseases (ARDs) in children can negatively impact renal function, potentially leading to acute kidney injury (AKI). This study compares the prevalence of AKI and other adverse in-hospital outcomes among hospitalized children with ARDs.

**Methods:**

A retrospective analysis was conducted using the United States Nationwide Inpatient Sample (NIS) database from 2005 to 2020. The study included children aged 1–17 years with ARDs, categorized into inflammatory arthritis, ANCA-associated vasculitis, systemic lupus erythematosus (SLE), systemic sclerosis (SSc), and other connective tissue diseases. Logistic regression assessed associations between ARD types and outcomes, including AKI, dialysis, and major adverse events.

**Results:**

Among 13,891 children with ARDs, 8.2% developed AKI and 1.3% required dialysis. Compared to inflammatory arthritis, ANCA-associated vasculitis significantly increased the risk of AKI (aOR = 11.20, 95% CI: 8.08–15.51) and dialysis (aOR = 40.60, 95% CI: 13.54-121.71). SLE also elevated risks of AKI (aOR = 4.16, 95% CI: 3.20–5.40) and dialysis (aOR = 11.34, 95% CI: 4.15–31.01). Children with SSc had increased risks of infection/pneumonia (aOR = 2.51, 95% CI: 1.84–3.41) and sepsis (aOR = 2.13, 95% CI: 1.26–3.58).

**Conclusions:**

Children with ARDs, especially those with ANCA-associated vasculitis and SLE, face elevated risks of AKI and dialysis. These findings underscore the importance of vigilant monitoring and tailored management in this population.

**Supplementary Information:**

The online version contains supplementary material available at 10.1186/s13052-025-01862-7.

## Introduction

Autoimmune rheumatic diseases (ARDs) are a heterogeneous group of chronic inflammatory disorders characterized by dysregulation of the immune system, resulting in the aberrant targeting of host tissues. These conditions can affect multiple organ systems, including, but not limited to the skin, eyes, lungs, heart, and blood vessels [1, 2]. The etiology of ARDs is multifactorial, involving complex interactions between genetic predisposition, environmental triggers, and immunological abnormalities. While the risk of developing ARDs generally increases with age due to cumulative exposure to potential triggers and age-related changes in immune function, certain subtypes, such as juvenile idiopathic arthritis (JIA), systemic lupus erythematosus (SLE), and antineutrophilic cytoplasmic antibody (ANCA)-associated vasculitis, frequently manifest in adolescence and childhood [3, 4].

ARDs have numerous systemic manifestations, and importantly can affect the kidneys. Renal involvement can result from various mechanisms, including immune complex deposition, vasculitis-induced ischemia, and medication-related nephrotoxicity, all of which can contribute to the development of chronic kidney disease (CKD) or acute kidney injury (AKI) [5–7]. AKI in children is a frequently overlooked complication, with serious impacts on both short- and long-term outcomes. In hospitalized children, AKI is associated with considerable morbidity and mortality, can lead to long-term health sequelae, and may potentially affect normal growth and development [8, 9].

The occurrence of ARDs and AKI in children is of particular concern due to several factors that elevate the risk profile. Children with chronic conditions, including ARDs, are at an increased risk of developing AKI due to various disease-related and treatment-related factors. These include the use of potentially nephrotoxic medications (such as nonsteroidal anti-inflammatory drugs, certain disease-modifying anti-rheumatic drugs, and some antimicrobials), the underlying inflammatory processes that can directly or indirectly affect renal function, and the potential for secondary infections that may precipitate or exacerbate kidney injury [10]. The complex interplay between these factors necessitates a comprehensive approach to patient management, and highlights the importance of regular monitoring of renal function in this high-risk group.

Thus, the purpose of this study was to comprehensively evaluate the outcomes of children hospitalized with ARDs, focusing on the frequency of AKI, and AKI requiring dialysis, and other major adverse inpatient outcomes such as infection and sepsis, using a nationally representative, large-scale dataset from the United States (US).

## Methods

### Study design and data source

This population-based, retrospective observational study extracted all data from the US Nationwide Inpatient Sample (NIS) database. The NIS is the largest all-payer, continuous inpatient care database in the United States, including about 8 million hospital stays each year. The database is administered by the Healthcare Cost and Utilization Project (HCUP) of the US National Institutes of Health (NIH). Patient data include primary and secondary diagnoses, primary and secondary procedures, admission and discharge status, patient demographic information, expected payment source, duration of hospital stay, and hospital characteristics (i.e., bed size/location/teaching status/hospital region). All admitted patients are initially considered for inclusion. The continuous, annually updated NIS database derives patient data from about 1,050 hospitals from 44 States in the US, representing a 20% stratified sample of US community hospitals as defined by the American Hospital Association.

### Ethics statement

This study complies with the terms of the NIS data-use agreement. All data were obtained through request to the Online Healthcare Cost and Utilization Project (HCUP) Central Distributor (available at: https://www.distributor.hcup-us.ahrq.gov/), which administers the database. Given that this study solely involved the analysis of secondary data, and there was no direct involvement of the general public or patients the requirement of informed patient consent was waived.

### Study population

This study utilized the International Classification of Diseases, Ninth and Tenth edition (ICD-9 and ICD-10) primary or secondary diagnostic codes to identify children between 1 and 17 years old who were admitted to US hospitals with a diagnosis of an ARD between 2005 and 2020 from the NIS database. Exclusion criteria were receiving dialysis but no diagnosis of AKI, missing information of covariates or main endpoints, and missing sample weights. The ARDs examined in this study were inflammatory arthritis (rheumatoid arthritis, juvenile idiopathic arthritis (JIA), ankylosing spondylitis, inflammatory spondyloarthropathy, and other inflammatory arthritis), anti-neutrophilic cytoplasmic antibody (ANCA)-associated vasculitis, systemic lupus erythematosus (SLE), systemic sclerosis (SSc), and other systemic connective tissue diseases. The detailed ICD codes for identifying the aforementioned conditions including the codes for the conditions excluded are summarized in Supplementary Table S1.

### Outcome measures

The outcomes studied were in-hospital mortality, AKI and AKI requiring dialysis, hospital length of stay (LOS), total hospital cost, and the occurrence of infection/pneumonia and sepsis. Since the NIS did not provide laboratory data, AKI and dialysis-requiring AKI were not identified using specific laboratory thresholds. Instead, they were identified through corresponding ICD-9 and ICD-10 diagnostic codes recorded in patients’ medical records, as documented in Supplementary Table S1. These diagnoses were made by U.S. clinicians based on their clinical judgment and applicable guidelines.

### Covariates

Patient characteristics included in the analysis were age categorized by age group (1–4, 5–9, 10–14, and 15–17 years), sex, race, household income, insurance status/primary payer, and admission type. Major comorbidities were identified using the ICD coding system, and included diabetes mellitus (DM), hypertension, ulcer or stomach problem, lung disease, and cancer. Hospital-related characteristics analyzed were bed size, location/teaching status, and hospital region. The ICD codes for identifying the comorbid conditions are summarized in Supplementary Table S1.

### Statistical analysis

Descriptive statistics were presented as number (n) and weighted percentage (%), or mean and standard error (SE). Categorical data were analyzed using the PROC SURVEYFREQ statement, which provides the Rao-Scott chi-square test for testing significance between weighted proportions. Continuous data were analyzed using the PROC SURVEYREG statement, which fits a linear model of the survey data and provides significance tests for model effects. Odds ratios (ORs), and 95% confidence intervals (CIs) were calculated for outcomes using logistic regression analysis. The Beta coefficient and 95% CI for LOS and total hospital cost were calculated using linear regressions. Covariates with significant differences between 2 comparison groups were adjusted for in multivariable regressions. All *p* values were 2-sided, and values of *p* < 0.05 were considered statistically significant. All statistical analyses were performed using SAS version 9.4 software (SAS Institute Inc., Cary, NC, US).

## Results

### Study population selection

**Fig. 1:**
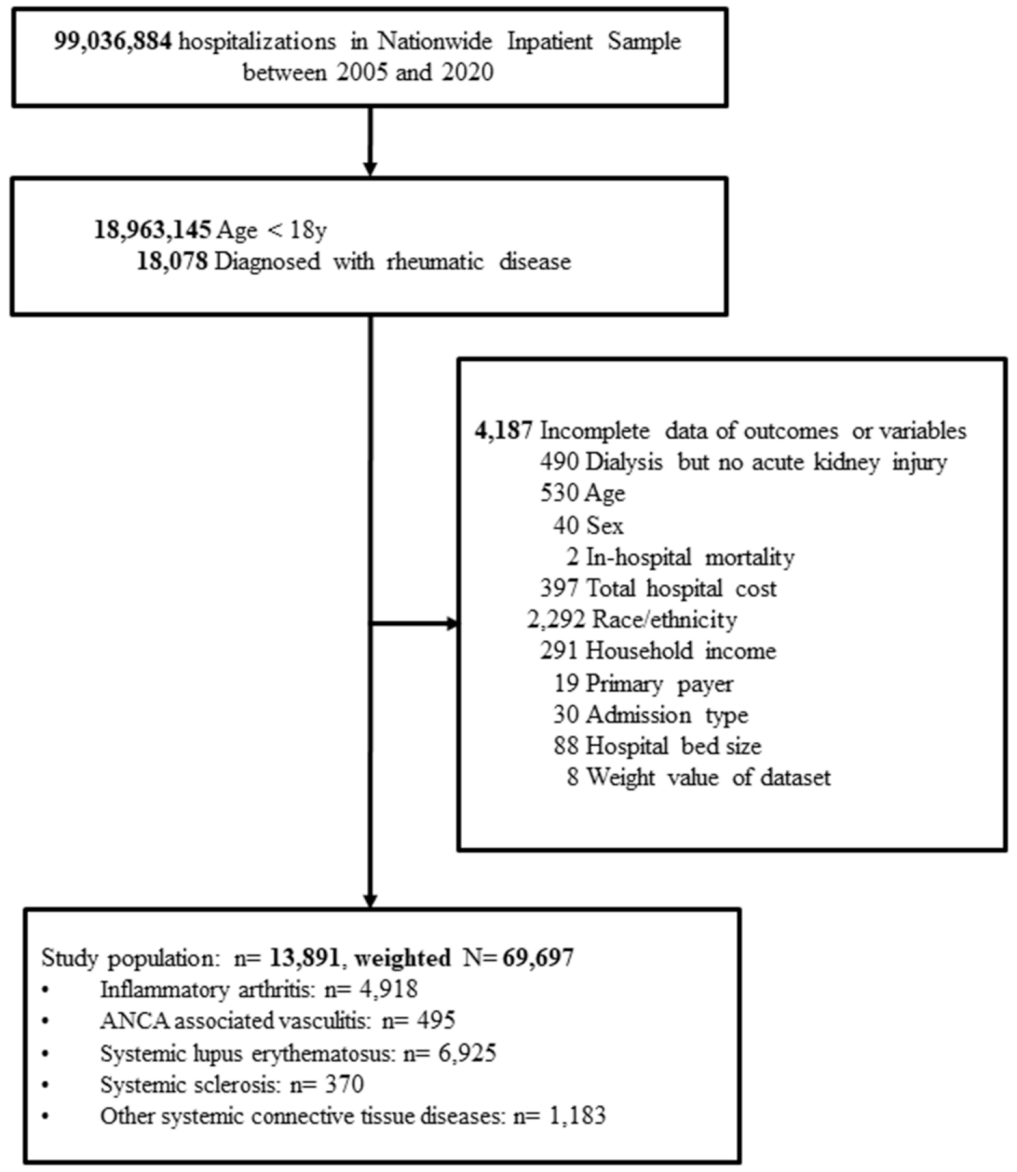
Flow diagram of patient selection process

The flow diagram of the patient selection process is shown in Fig. 1. A total of 18,078 hospitalized children < 18 years old who were diagnosed with an ARD were identified in the NIS from 2005 to 2020. Children with missing information on study outcomes or variables (*n* = 4,187) were excluded, and thus 13,891 children were included in the analyses. Of the 13,891 children, 4,918 (35.4%) had inflammatory arthritis, 495 (3.7%) had ANCA-associated vasculitis, 6,925 (49.9%) had SLE, 370 (2.7%) had SSc, and 1,183 (8.5%) had other systemic connective tissue diseases. This sample can be extrapolated to a total of 69,697 children in the entire US after applying the weight values provided by the dataset (Fig. 1).

### Characteristics of children hospitalized with ARDs

Characteristics of the study population are summarized in Table 1. The mean age of the children was 12.8 years, most were female (72%), 38% were White, 31% were in quartile 1 of household income, 49% had insurance covered by Medicare or Medicaid, and 79% were admitted emergently. A large percentage of the children were admitted to large hospitals (65%), urban teaching hospitals (88%), and hospitals located in the Midwest (41%). The most common comorbidity was hypertension (17%), and 69% of the children had a Charlson Comorbidity Index (CCI) of 0 points. (Table 1)

**Table 1:** Characteristics of hospitalized children with ARDs

	Total	Inflammatory arthritis	ANCA-associated vasculitis	SLE	SSc	Other systemic connective tissue diseases	*p* value
	(*N* = 13,891)	(*n* = 4,918)	(*n* = 495)	(*n* = 6,925)	(*n* = 370)	(*n* = 1,183)	
In-hospital mortality	56 (0.4)	15 (0.3)	4 (0.8)	24 (0.3)	5 (1.3)	8 (0.7)	**0.009**
Acute kidney injury	1,134 (8.2)	89 (1.8)	127 (25.5)	797 (11.6)	11 (3.0)	110 (9.3)	**< 0.001**
AKI requiring dialysis	186 (1.3)	4 (0.1)	33 (6.7)	143 (2.1)	2 (0.5)	4 (0.3)	**< 0.001**
Length of stay, days	5.5 ± 0.1	4.9 ± 0.1	8.5 ± 0.6	5.5 ± 0.1	5.7 ± 0.5	6.5 ± 0.3	**< 0.001**
Total hospital cost, ×1,000 USD	58.5 ± 1.7	38.9 ± 1.1	137.4 ± 18.2	57.5 ± 2.1	61.4 ± 7.3	110.2 ± 8.7	**< 0.001**
Infection/pneumonia	1,522 (11.0)	448 (9.1)	70 (14.4)	798 (11.5)	75 (20.4)	131 (11.1)	**< 0.001**
Sepsis	1,086 (7.8)	411 (8.4)	52 (10.5)	418 (6.0)	60 (16.1)	145 (12.3)	**< 0.001**
Age, years	12.8 ± 0.04	11.7 ± 0.08	13.8 ± 0.16	14.0 ± 0.05	13.1 ± 0.21	10.5 ± 0.15	**< 0.001**
1–4	829 (6.0)	538 (10.9)	7 (1.4)	70 (1.0)	10 (2.8)	204 (17.3)	**< 0.001**
5–9	1,690 (12.2)	903 (18.4)	45 (9.0)	451 (6.5)	38 (10.4)	253 (21.5)	
10–14	5,038 (36.3)	1,576 (32.1)	179 (36.2)	2,731 (39.4)	164 (44.2)	388 (32.8)	
15–17	6,334 (45.6)	1,901 (38.7)	264 (53.4)	3,673 (53.1)	158 (42.6)	338 (28.4)	
Sex							**< 0.001**
Male	3,875 (27.9)	1,768 (36.1)	233 (46.9)	1,251 (18.1)	94 (25.3)	529 (44.6)	
Female	10,016 (72.1)	3,150 (63.9)	262 (53.1)	5,674 (81.9)	276 (74.7)	654 (55.4)	
Race							**< 0.001**
White	5,295 (38.2)	2,893 (58.9)	323 (65.4)	1,374 (19.9)	157 (42.6)	548 (46.2)	
Black	3,500 (25.1)	690 (14.0)	51 (10.3)	2,461 (35.3)	60 (16.2)	238 (20.1)	
Hispanic	3,516 (25.3)	922 (18.7)	84 (17.0)	2,098 (30.3)	129 (34.6)	283 (24.0)	
Others	1,580 (11.4)	413 (8.4)	37 (7.3)	992 (14.4)	24 (6.7)	114 (9.7)	
Household income							**< 0.001**
Quartile1	4,349 (31.3)	1,249 (25.5)	131 (26.2)	2,497 (36.0)	118 (31.8)	354 (30.1)	
Quartile2	3,238 (23.3)	1,201 (24.3)	89 (18.1)	1,575 (22.7)	86 (23.3)	287 (24.4)	
Quartile3	3,223 (23.2)	1,237 (25.1)	121 (24.5)	1,519 (22.0)	90 (24.5)	256 (21.4)	
Quartile4	3,081 (22.2)	1,231 (25.1)	154 (31.3)	1,334 (19.3)	76 (20.4)	286 (24.2)	
Primary payer							**< 0.001**
Medicare/Medicaid	6,808 (49.1)	2,046 (41.6)	186 (37.6)	3,900 (56.4)	135 (36.8)	541 (45.8)	
Private including HMO	6,015 (43.3)	2,530 (51.5)	283 (57.3)	2,458 (35.5)	171 (45.9)	573 (48.4)	
Self-pay/no-charge/other	1,068 (7.6)	342 (6.9)	26 (5.2)	567 (8.1)	64 (17.3)	69 (5.8)	
Admission type							**< 0.001**
Emergent	10,982 (79.1)	4,006 (81.5)	388 (78.4)	5,353 (77.3)	249 (67.6)	986 (83.4)	
Elective	2,909 (20.9)	912 (18.5)	107 (21.6)	1,572 (22.7)	121 (32.4)	197 (16.6)	
Hospital bed size							**0.008**
Small	1,765 (12.3)	618 (12.1)	45 (8.9)	856 (12.0)	58 (15.5)	188 (15.9)	
Medium	3,101 (22.8)	1,053 (21.8)	108 (22.3)	1,550 (22.8)	82 (22.5)	308 (26.3)	
Large	9,025 (64.9)	3,247 (66.1)	342 (68.8)	4,519 (65.2)	230 (62.0)	687 (57.9)	
Location/teaching status							**< 0.001**
Rural	373 (2.7)	216 (4.4)	9 (1.8)	129 (1.9)	4 (1.0)	15 (1.2)	
Urban nonteaching	1,267 (8.9)	687 (13.6)	28 (5.6)	466 (6.6)	20 (5.2)	66 (5.5)	
Urban teaching	12,251 (88.4)	4,015 (82.0)	458 (92.5)	6,330 (91.5)	346 (93.8)	1,102 (93.3)	
Hospital region							**< 0.001**
Northeast	2,744 (19.9)	1,059 (21.7)	111 (22.4)	1,301 (18.9)	55 (15.0)	218 (18.4)	
South	2,003 (14.5)	800 (16.4)	63 (12.7)	809 (11.7)	58 (15.8)	273 (23.0)	
Midwest	5,816 (41.4)	2,019 (40.5)	178 (35.6)	3,032 (43.4)	135 (36.2)	452 (38.1)	
West	3,328 (24.2)	1,040 (21.3)	143 (29.3)	1,783 (26.0)	122 (33.0)	240 (20.5)	
Major comorbidities							
Diabetes mellitus	363 (2.6)	200 (4.1)	11 (2.2)	106 (1.5)	10 (2.7)	36 (3.0)	**< 0.001**
Hypertension	2,434 (17.5)	222 (4.5)	109 (22.1)	1,961 (28.3)	52 (14.0)	90 (7.6)	**< 0.001**
Myocardial infarction	16 (0.1)	2 (0.0)	1 (0.2)	8 (0.1)	0 (0.0)	5 (0.4)	-
Ulcer or stomach problem	543 (3.9)	245 (5.0)	12 (2.5)	218 (3.2)	31 (8.4)	37 (3.1)	**< 0.001**
Lung disease	2,063 (14.9)	838 (17.0)	77 (15.9)	805 (11.7)	129 (34.8)	214 (18.0)	**< 0.001**
Cancer	182 (1.3)	86 (1.7)	4 (0.8)	58 (0.8)	22 (6.0)	12 (1.0)	**< 0.001**
Charlson Comorbidity Index							**< 0.001**
0	9,602 (69.1)	3,600 (73.2)	266 (53.7)	4,691 (67.8)	231 (62.7)	814 (68.9)	
1	2,390 (17.2)	1,000 (20.3)	70 (14.2)	938 (13.6)	99 (26.5)	283 (23.9)	
2	1,463 (10.5)	232 (4.7)	135 (27.1)	1,011 (14.5)	23 (6.2)	62 (5.2)	
3	339 (2.5)	61 (1.3)	22 (4.6)	226 (3.3)	12 (3.3)	18 (1.5)	
4+	97 (0.7)	25 (0.5)	2 (0.4)	59 (0.9)	5 (1.3)	6 (0.5)	

Abbreviations: ARDs, autoimmune rheumatic diseases; ANCA, anti-neutrophilic cytoplasmic antibody; AKI, acute kidney injury; HMO, Health Maintenance Organization; CCI, Charlson Comorbidity Index. SLE, systemic lupus erythematosus; SSc, systemic sclerosis

Continuous variables are presented as mean ± SE; categorical variables are presented as unweighted counts (weighted percentage)

*p* values < 0.05 are shown in bold

### In-hospital outcomes between different type of ARDs

Children with ANCA-associated vasculitis had the highest percentages of AKI (25.5%) and AKI requiring dialysis (6.7%) (*p* < 0.001). These children also had the longest LOS (8.5 days), and greatest total hospital costs (137.4 thousand USD) (*p* < 0.001). On the other hand, children with SSc had the highest percentages of in-hospital mortality (1.3%, *p* = 0.009), infection/pneumonia (20.4%, *p* < 0.001), and sepsis (16.1%, *p* < 0.001) among the groups. (Table 1)

### Associations between type of ARDs, in-hospital mortality, AKI, dialysis, infection/pneumonia, and sepsis

The associations between types of ARDs and in-hospital mortality, AKI, dialysis, infection/pneumonia, and sepsis are shown in Tables 2 and 3. After adjustment, the multivariable analysis revealed that compared to inflammatory arthritis, children with ANCA-associated vasculitis had increased risks of AKI (aOR [adjusted OR] = 11.20, 95% CI: 8.08–15.51), AKI requiring dialysis (aOR = 40.60, 95% CI: 13.54-121.71), and infection/pneumonia (aOR = 1.79, 95% CI: 1.30–2.45).

**Table 2:** Associations between type of ARDs and in-hospital mortality and AKI

Type of ARDs	In-hospital mortality ^a^	AKI ^b^	AKI requiring dialysis ^c^
	aOR (95% CI)		
Inflammatory arthritis	Ref.	Ref.	Ref.
ANCA associated vasculitis	1.61 (0.46, 5.59)	**11.20 (8.08**, **15.51)**	**40.60 (13.54**, **121.71)**
SLE	0.81 (0.37, 1.76)	**4.16 (3.20**, **5.40)**	**11.34 (4.15**, **31.01)**
SSc	2.86 (0.94, 8.72)	1.20 (0.64, 2.24)	3.94 (0.75, 20.59)
Other systemic connective tissue diseases	2.02 (0.84, 4.83)	**5.11 (3.77**, **6.91)**	3.29 (0.82, 13.15)

Abbreviations: ARDs, autoimmune rheumatic diseases; ANCA, anti-neutrophilic cytoplasmic antibody; AKI, acute kidney injury; OR, odds ratio; CI, confidence interval; ref, reference; SLE, systemic lupus erythematosus; SSc, systemic sclerosis

Multivariable regression was adjusted for variables that were significant in the univariate regression model

*p* values < 0.05 are shown in bold

^a^ Adjusted for admission type, location/teaching status, hypertension, myocardial infarction, cancer, and CCI

^b^ Adjusted for age group, sex, race, household income, primary payer, admission type, location/teaching status, hospital region, hypertension, and CCI

^c^ Adjusted for age group, race, primary payer, admission type, hospital region, hypertension, myocardial infarction, and CCI

**Table 3:** Associations between type of ARDs and infection/pneumonia and sepsis

Type of ARDs	Infection/Pneumonia ^a^	Sepsis ^b^
	aOR (95% CI)	
Inflammatory arthritis	Ref.	Ref.
ANCA associated vasculitis	**1.79 (1.30**, **2.45)**	1.26 (0.91, 1.74)
SLE	**1.48 (1.27**, **1.72)**	**0.72 (0.61**, **0.84)**
SSc	**2.51 (1.84**, **3.41)**	**2.13 (1.26**, **3.58)**
Other systemic connective tissue diseases	1.18 (0.95, 1.46)	**1.38 (1.08**, **1.78)**

Abbreviations: ARDs, autoimmune rheumatic diseases; ANCA, anti-neutrophilic cytoplasmic antibody; AKI, acute kidney injury; OR, odds ratio; aOR, adjusted odds ratio; CI, confidence interval; Ref., reference; SLE, systemic lupus erythematosus; SSc, systemic sclerosis

Multivariable regression was adjusted for variables that were significant in the univariate regression model

*p* values < 0.05 are shown in bold

^a^ Adjusted for age group, sex, race, household income, primary payer, admission type, hypertension, lung disease, cancer, and CCI

^b^ Adjusted for age group, sex, household income, primary payer, admission type, myocardial infarction, cancer, and CCI

Compared with inflammatory arthritis, after adjustment, children with SSc had increased risks of infection/pneumonia (aOR = 2.51, 95% CI: 1.84–3.41) and sepsis (aOR = 2.13, 95% CI: 1.26–3.58). Compared with inflammatory arthritis, after adjustment children with SLE had higher risks of AKI (aOR = 4.16, 95% CI: 3.20–5.40), AKI requiring dialysis (aOR = 11.34, 95% CI: 4.15–31.01), and infection/pneumonia (aOR = 0.48, 95% CI: 1.27–1.72), but a lower risk of sepsis (aOR = 0.72, 95% CI: 0.61–0.84).

Compared with inflammatory arthritis, after adjustment children with other systemic connective tissue diseases had a higher risk of AKI (aOR = 5.11, 95% CI: 3.77–6.91) and sepsis (aOR = 1.38, 95% CI: 1.08–1.78). (Tables 2 and 3)

### Associations between type of ARDs, LOS, and total hospital costs

The associations between types of ARDs, LOS, and total hospital costs are summarized in Table 4. Compared with inflammatory arthritis, children with ANCA-associated vasculitis or other systemic connective tissue disease had longer LOS (aBeta = 2.42 and 1.20, respectively) compared to other groups. In addition, children who had ANCA-associated vasculitis (81.42 thousand USD), SLE (6.22 thousand USD), or other systemic connective tissue disease (65.68 thousand USD) had significantly greater total hospital costs as compared to those with inflammatory arthritis. (Table 4)

**Table 4:** Associations between type of ARDs and LOS and total hospital costs

Type of ARD	LOS ^a^	Total hospital costs ^b^
	aBeta (95% CI)	
Inflammatory arthritis	Ref.	Ref.
ANCA associated vasculitis	**2.42 (1.36**, **3.48)**	**81.42 (47.26**, **115.58)**
SLE	-0.29 (-0.63, 0.06)	**6.22 (0.80**, **11.63)**
SSc	0.30 (-0.52, 1.12)	11.31 (-2.62, 25.24)
Other systemic connective tissue diseases	**1.20 (0.59**, **1.81)**	**65.68 (49.53**, **81.83)**

Abbreviations: ARDs, autoimmune rheumatic diseases; ANCA, anti-neutrophilic cytoplasmic antibody; CI, confidence interval; Ref., reference; SLE, systemic lupus erythematosus; SSc, systemic sclerosis; LOS, length of stay

Multivariable regression was adjusted for variables that were significant in the univariate regression model

*p* values < 0.05 are shown in bold

^a^ Adjusted for sex, race, primary payer, admission type, location/teaching status, hospital region, hypertension, ulcer or stomach problem, lung disease, cancer, and CCI

^b^ Adjusted for sex, race, admission type, location/teaching status, hospital region, hypertension, ulcer or stomach problem, cancer, and CCI

## Discussion

This study analyzed data from more than 10 thousand hospitalized children with ARDs in the US, revealing distinct patterns of complications and healthcare utilization patterns across different type of ARDs. To the best of our knowledge, this is the first study to comprehensively compare inpatient outcomes of children with various ARDs using a national database. Our findings indicate varied risks of unfavorable inpatient outcomes among different types of ARDs. The prevalence of AKI was highest in children with ANCA-associated vasculitis (25%), and in those with AKI requiring dialysis (7%). Children with SLE had an AKI frequency of 12%, and 2% had AKI requiring dialysis. Additionally, the frequency of sepsis was > 10% in children with ANCA-associated vasculitis, SSc, and other systemic connective tissue diseases. After adjusting for demographic, clinical, and hospital-related characteristics, compared to inflammatory arthritis, children with ANCA-associated vasculitis had the highest risks of unfavorable hospitalization outcomes, including AKI and AKI requiring dialysis, and infection/pneumonia. Compared to children with inflammatory arthritis, children with SLE also had significantly greater risks for AKI and AKI requiring dialysis, and infections. While children with SSc did not have a significantly higher risk of AKI, they had higher risks of pneumonia/infections and sepsis compared to children with inflammatory arthritis. Furthermore, ANCA-associated vasculitis was strongly associated with longer LOS and significantly increased total hospital costs compared to inflammatory arthritis. These findings underscore the need for tailored management strategies and vigilant monitoring of children with ARDs, especially ANCA-associated vasculitis and SLE, to mitigate the higher risks of severe complications and optimize healthcare resource utilization.

While ARDs are not common in children, they can have the same serious and life-threatening complications and outcomes such as AKI as in adults. Vasculitis is common to many ARDs, which can cause small- or medium-vessel inflammation and is commonly associated with acute glomerulonephritis (AGN) that can lead to AKI or end-stage renal disease (ESRD) when not treated [11]. Further, ARDs might be associated with the formation of immune complexes that can deposit in renal tissues, triggering inflammation and contributing to glomerulonephritis [12]. This process might lead to AKI, especially during disease flares or when ARD activity is high. On the other hand, patients with ARDs often require immunosuppressive therapies, non-steroidal anti-inflammatory drugs (NSAIDs), and other medications that can have nephrotoxic effects [13]. Further, glucocorticoids are one of the primary treatments for ARDs, and the side effects can be more serious in children than in adults because the medications disrupt the normal functioning of the endocrine system which of vital for proper growth and development [14]. Notably, in adults with ARDs kidney damage tends to be of slow onset and chronic with gradual loss of renal function, whereas in children kidney damage tends to be an acute inflammatory process and kidney damage is more likely to be irreversible, even with prompt treatment [15]. Kidney transplantation is an option for children with an ARD who develop kidney failure; however, it has been documented that posttransplant patient and allograft outcomes are worse in children with SLE than in those with other ARDs [16]. Notably, childhood obesity, a significant public health issue with alarming rates in the developed world, is a recognized risk factor for various childhood-onset ARDs in the pediatric population. It has both pathophysiological and clinical implications for ARDs treatment responses [17].

Our results suggest that, among US pediatric population, ANCA-associated vasculitis is associated with the most adverse inpatients outcomes, including a higher risk of AKI and AKI requiring dialysis, infection/pneumonia, and longer LOS. These results are consistent with those of prior studies specifically focusing on pediatric ANCA-associated vasculitis. A review by Plumb et al. [18] concluded that the course of ANCA-associated vasculitis is more severe in children than in adults, and children are much more like to have renal involvement at presentation. Importantly, a large percentage of children progress to ESRD despite prompt treatment. Zhang et al. [19] studied 24 children with ANCA-associated vasculitis admitted to their hospital, and examined the effect of serum complement C3 levels on disease course. Children were divided into a low C3 group and a normal C3 group, and the analysis showed that the outcomes of children with a low C3 level were markedly worse than those with a normal level including higher erythrocyte sedimentation rate (ESR), higher serum creatinine, worse histological changes on renal biopsy, and greater C3 renal deposition. In the low C3 group 3 of the children progressed to ESRD, and 1 child died. In a somewhat similar study, Charnaya et al. [20] examined the characteristics of 17 children (median age 15 years) with biopsy proven ANCA-associated glomerulonephritis. During treatment, 1 child died and 6 progressed to kidney failure. Proteinuria at presentation and glomerulosclerosis and interstitial fibrosis and tubular atrophy (IFTA) identified on renal biopsy were risk factors for kidney failure and death. These findings, along with our observations, underscore the critical need for improving care strategies for ANCA-associated vasculitis in the pediatric population.

One of the most common ARDs in adults is SLE, and it was the most common in our pediatric study population. As with adults, our results showed that children with SLE were at high risk for AKI, AKI requiring dialysis, and infections. It has been reported that up to 15% of children with SLE progress to ESRD [21, 22]. Notably, vasculitis is an important component of SLE in children and symptoms can be non-specific and overlap with those of SLE, and can be life threatening if multiple organ systems are involved [23]. Immunosuppression is the primary treatment of SLE in adults and children; however, the complete response rate in children is only 40–60% [22]. Cann et al. [24] studied 42 children with SLE (average age at diagnosis 12.5 years): 29 had renal involvement and of 27 children with biopsy proven lupus nephritis (LN) 70% had Class III or IV disease. Few studies have examined the long-term outcomes of SLE that presents in childhood. Mirquet et al. [25] looked at the outcomes of 138 children with a median follow-up of 15 years. Half of the patients had a Systemic Lupus International Collaborating Clinics (SLICC) Damage Index score ≥ 1 at last follow-up, with the musculoskeletal, cutaneous, neurological, cardiovascular, and renal damage being the most common manifestations. A 2017 US study [26] using Medicare claims reported that hospitalized infections were very common in children with SLE, with bacterial pneumonia being the most prevalent, occurring at a rate of 10.42 per 100 person-years among all SLE patients. The highest infection risks were observed in African American and American Indian children, those with LN, comorbidities, and those taking corticosteroids. Another recent study documented that high disease activity, lupus nephritis (LN), and lymphopenia were predictors of major infections in newly diagnosed childhood-onset SLE patients [27]. These findings support our observations regarding the infection risk in childhood SLE.

Our results indicated that US children with SSc have higher risks of infection, pneumonia, and sepsis compared to those with inflammatory arthritis. This is the first report in the literature to document these findings. Childhood SSc is considered a very rare orphan disease with very few publications regarding the condition [28]; and among the ARDs we focused, SSc is much less common in children that the other conditions we examined. Beukelman et al. [28] reviewed claims data and estimated the prevalence of juvenile SSc to be about 3 cases per 1 million children. A review by Li (2018) [29] collected evidence on juvenile SSc and localized scleroderma, documenting that morbidity and mortality are significantly worse for juvenile SSc. Patients are at risk for life-threatening fibrosis of the lungs, heart, and other visceral organs, as well as vasculopathy. The review also indicated that early diagnosis can greatly improve outcomes.

In the present study, we lumped all inflammatory arthritis together, which included JIA. JIA is defined as arthritis of unknown origin lasting over six weeks with onset before age 16. It is the most common ARD in children [30]. Gicchino et al. [31] examined the records of 110 children with JIA with a median age at the last follow-up visit of 14 years. The overall results showed that about 8% of the children developed hypertension or CKD, and the main risk factors were longer exposures to both non-steroidal anti-inflammatory drugs (NSAIDs) and methotrexate due to more severe disease. Cafarotti et al. [32] compared renal function indices between 49 children with JIA (mean age 10 years) with those of 49 healthy control children (mean age 11 years). Cystatin C (CysC) and blood urea nitrogen (BUN) levels were significantly higher and creatine and eGFR were significantly lower in the children with JIA compared to the control children. The renal resistive index (RRI), a sonographic index of blood flow in the renal artery with higher values associated with renal disease and poorer outcomes, was significantly greater in the children with JIA compared with the control children. Although the risk of AKI in children with inflammatory arthritis appears the lowest compared to other ARDs in our analysis, significant renal complications still occur in children with JIA according to the above cited studies, highlighting the need for careful monitoring.

### Clinical implications

The findings of this study highlight important clinical implications for managing hospitalized children with ARDs in the US Children with ANCA-associated vasculitis and SLE face high risks of AKI and dialysis, necessitating vigilant renal monitoring and early intervention. Tailored treatment plans should address these specific risks, including careful use of nephrotoxic medications. Children with SSc have higher risks of infection, pneumonia, and sepsis, suggesting the need for enhanced infection control measures. Additionally, the longer hospital stays and higher costs for children with ANCA-associated vasculitis call for efficient resource allocation and management strategies to optimize healthcare utilization.

### Strengths and limitations

One of the main strengths of this study is its use of a large, nationally representative database, the NIS, which enhances the generalizability and robustness of the findings. This comprehensive dataset allowed us to capture a wide range of conditions and outcomes, including rare occurrences, providing a detailed and reliable analysis of inpatient outcomes among children with different ARDs. A key limitation of the study is its retrospective design, which relies on administrative data and ICD codes for identifying ARD diagnoses and outcomes. This reliance on secondary data may introduce potential misclassification or coding errors, and it does not allow for the assessment of clinical details or patient-reported outcomes. Additionally, the retrospective nature may introduce selection bias. Disease status, such as disease activity, cannot be analyzed because the NIS database lacks information on laboratory measurements. Disease-modifying drugs are crucial in the management of ARDs, and affect the outcomes evaluated; however, detailed medication use and adherence, which could influence the development and progression of complications like AKI, are not available in the NIS. Lastly, potential long-term sequelae, such as progression to CKD, are clinically critical considerations relevant to the present investigations. However, the lack of follow-up information in the dataset limits our ability to analyze these outcomes. Future studies are needed to address this important issue comprehensively.

## Conclusion

This study, analyzing data from over 10,000 hospitalized children with ARDs in the US, revealed distinct patterns of complications and healthcare utilization across different types of ARDs. The findings indicate varied risks of unfavorable inpatient outcomes, with ANCA-associated vasculitis and SLE showing the highest risks for AKI and related complications. These results highlight the need for tailored management strategies and vigilant monitoring, particularly for children with ANCA-associated vasculitis and SLE, to reduce severe complications and optimize healthcare resource utilization.

## Electronic supplementary material

Below is the link to the electronic supplementary material.

Supplementary Material 1

## Data Availability

All of the data supporting underlying findings are included in the manuscript.
