# Supplementary material for: Acute kidney injury and outcomes in hospitalized children with autoimmune rheumatic disease
Source: Ital J Pediatr. 2025 Feb 7;51:37. doi: 10.1186/s13052-025-01862-7 (PMC11806621; doi:10.1186/s13052-025-01862-7)
Supplement: Supplementary file 1 — Supplementary Material 1 [file 13052_2025_1862_MOESM1_ESM.docx]

**Supplementary Table 1. ICD codes used in this study**

|  | ICD-9-CM | ICD-9-PCS | ICD-10-CM | ICD-10-PCS |
| --- | --- | --- | --- | --- |
| ARDs |  |  |  |  |
| Inflammatory arthritis | 714, 720.0, 720.2-720.9 |  | M05-M06, M45.9, M46.1, M46.80, M46.90, M49.80 |  |
| ANCA associated vasculitis | 446.3-446.4 |  | M30.1, M31.2-M31.3 |  |
| SLE | 710.0 |  | M32 |  |
| SSc | 710.1 |  | M34 |  |
| Other systemic connective tissue diseases | 710.5, 710.9 |  | M35.1, M35.4, M35.8, M35.9 |  |
| AKI | 584 |  | N17 |  |
| Dialysis-requiring AKI | V45.11, V56.0, V56.1 | 39.95 | Z49.01, Z49.31, Z99.2 | 5A1D |
| Infection/pneumonia | 073.0, 115.15, 115.95, 480, 481, 482, 483, 484.7, 484.8, 485, 486, 514, 517, 680-686, 695.1, 695.81, 91[0-2].[1, 3, 5, 7, 9] |  | J12-J18, L00-L08 |  |
| Sepsis | 995.9, 996.64, 38, 999.3, 790.7, 41, 785.52 |  | R78.81, A41, R65.2, T81.4, T80.2, A42.7, A22.7, B37.7, A26.7, A28.2, A54.86, B00.7, A32.7, A24.1, A39.2, A20.7, A21.7, A48.3 |  |
| DM | 250 |  | E10-E13 |  |
| Hypertension | 401, 405 |  | I10, I15, I16 |  |
| Ulcer or stomach problem | 531-535.7, 536.3, 536.8-536.9, 578.9 |  | K25-K30, K31.84, K31.89, K31.9, K52.81, K92.2 |  |
| Lung disease | 490-493, 494-496, 500-505, 515-517, 714.81 |  | J40-J47, J60-J67, J84, J99, M05.10-M05.19 |  |
| Cancer | 140-209.39 |  | C00-C26, C30-C41, C43-C58, C60-C75, C76-C86, C88.2-C88.9, C90-C93, C94.0-C94.3, C94.8, C95-C96, C7A |  |
